# Formulation of Agarose Gels Containing Chitosan-Oleic Acid Complex Particles and Their Physical and In Vitro Digestion Properties

**DOI:** 10.3390/gels12050374

**Published:** 2026-04-29

**Authors:** Takashi Kuroiwa, Tsukasa Kikuchihara, Kana Kanemitsu, Airi Kato

**Affiliations:** 1 Department of Applied Chemistry, Faculty of Science and Engineering, Tokyo City University, 1-28-1 Tamazutsumi, Setagaya-ku, Tokyo 158-8557, Japan; 2 Advanced Research Laboratories, Tokyo City University, 8-15-1 Todoroki, Setagaya-ku, Tokyo 158-0082, Japan

**Keywords:** composite hydrogel, encapsulation, hydrophobic bioactive, curcumin, human gastric digestion simulator

## Abstract

In this study, we developed new food gel materials, prepared agarose (AG) gels containing chitosan (CHI)-oleic acid (OA) complex particles, and evaluated their structure, mechanical properties, and in vitro digestion characteristics. CHI-OA complex particles, with an average diameter of approximately 0.9 mm, were successfully incorporated into 1–3 wt% AG gels by mixing with an aqueous AG solution and cooling it while maintaining a uniform dispersion state of the complex particles after gelation. The incorporation of CHI-OA complex particles affected the gelation behavior of AG during cooling and altered the mechanical properties of the resulting gel. The digestion properties of the CHI-OA-AG gel were evaluated through in vitro gastric digestion experiments using a flask shaker and a human gastric digestion simulator. After 120 min of flask shaking, the CHI-OA-AG gel maintained its shape, whereas significant disintegration and fragmentation were observed after 120 min in the human gastric digestion simulator. Notably, most CHI-OA complex particles were retained within the gel fragments even after disintegration, with <5% of the total particles released into the simulated gastric juice. In addition, we prepared a CHI-OA-AG gel encapsulating water-insoluble curcumin (CUR) using the hydrophobic domains of the CHI-OA complex particles. CUR was successfully incorporated into the gel at concentrations up to 72 μmol/L, suggesting that CUR contained in the CHI-OA-AG dispersion before gelation was completely encapsulated. These results demonstrate the potential applicability of the CHI-OA-AG composite gel as a next-generation food material with enhanced nutritional value and controlled digestibility.

## 1. Introduction

Water-insoluble bioactive compounds in foods, such as hydrophobic polyphenols and oil-soluble vitamins, have various beneficial effects on health, including the prevention of cardiovascular diseases, brain diseases, and cancer [1,2]. When using these hydrophobic bioactives, their low water solubility makes them unabsorbable in the body; that is, their low bioavailability is problematic [3,4]. Therefore, technologies for delivering hydrophobic bioactive compounds using appropriate carriers are necessary to promote the development of value-added food products that take advantage of their health effects. Recently, various delivery methodologies using micelles, emulsions, liposomes, molecular complexes, lipid nanoparticles, and hydrogels have been investigated [5,6,7,8,9,10,11,12,13,14].

The development of foods that enable these delivery technologies is important for people experiencing difficulty in nutrient intake from their regular diet, such as children, the elderly, or those with digestive disorders. It is possible to ingest nutrients through drinkable liquid foods and swallowable tablet supplements. However, physical functions related to eating, such as mastication, swallowing, and digestion, are maintained through eating behaviors [15]. Therefore, it is important to develop solid/semi-solid food materials that provide nutrition and can be taken with the experience of eating. From this perspective, it is desirable to develop food materials that can deliver functional hydrophobic nutritional ingredients orally to people in various life stages and health conditions.

The design methodology for solid/semi-solid foods with various textures is attracting attention when considering foods for the elderly and patients with digestive disorders. In addition, among these, hydrogels are solid food materials with excellent processability. Their mechanical properties, such as hardness and elasticity, can be controlled by their constituent components and preparation conditions. Hydrogel-based nutrient delivery systems have been intensely studied [15,16,17,18,19,20]. However, one technical issue requiring further development is the retention and controlled release of hydrophobic nutritional components. Methods for retaining hydrophobic components in hydrogels include the use of emulsions, solid particles, liposomes, and molecular complexes [15,21,22]. While emulsion-based hydrogels are widely used, they require the incorporation of lipid phases, which may not always be desirable from a nutritional perspective, and the encapsulated oil phase may be destabilized or released during digestion. Other approaches, such as liposomes or particle-based systems, often involve relatively complex preparation procedures and may limit the loading efficiency of hydrophobic compounds. In addition, the relationship between gel structure and digestive behavior has not always been sufficiently clarified. Therefore, there is a need to develop new hydrogel materials and to evaluate their properties as delivery carriers, including mechanical properties, digestibility, and encapsulation ability.

In this study, we focused on the use of chitosan (CHI)-oleic acid (OA) complex particles [23] as a new approach for incorporating hydrophobic bioactives into hydrogels. CHI-OA complex particles are submicron-sized particles formed by the spontaneous complexation of CHI, a cationic polysaccharide composed of D-glucosamine, and OA, a fatty acid with an unsaturated long alkyl chain. The OA association structure formed within the particles allows hydrophobic components to be retained within the particles. These particles successfully disperse hydrophobic curcumin (CUR, LogP = ~3), capsaicin (LogP = 3.2), limonene (LogP = 4.58), and α-mangostin (LogP = 7.7) in aqueous media using CHI-OA complex particles [23,24,25,26]. Hydrophobic components can be incorporated into the hydrogel by entrapping these CHI-OA particles in a hydrogel matrix. Owing to its wide use in food and biotechnology, agarose (AG) was used as the gel material in this study. AG, the main component of agar derived from seaweed, is a neutral polysaccharide composed of a chemical structure that repeats agarobiose units of β-D-galactose and 3,6-anhydro-L-galactose [27]. We prepared AG hydrogels containing CHI-OA complex particles by simply mixing and cooling the CHI-OA complex particle dispersion in the AG solution. In addition to characterizing their structure and mechanical properties, we evaluated their digestibility through in vitro digestion tests using a human gastric digestion simulator [28,29] in accordance with the INFOGEST protocol [30]. Furthermore, we demonstrated that hydrogels containing CUR could be prepared using CHI-OA complex particles encapsulating CUR.

## 2. Results and Discussion

### 2.1. Preparation of AG Gels Containing CHI-OA Complex Particles

**Figure 1:**
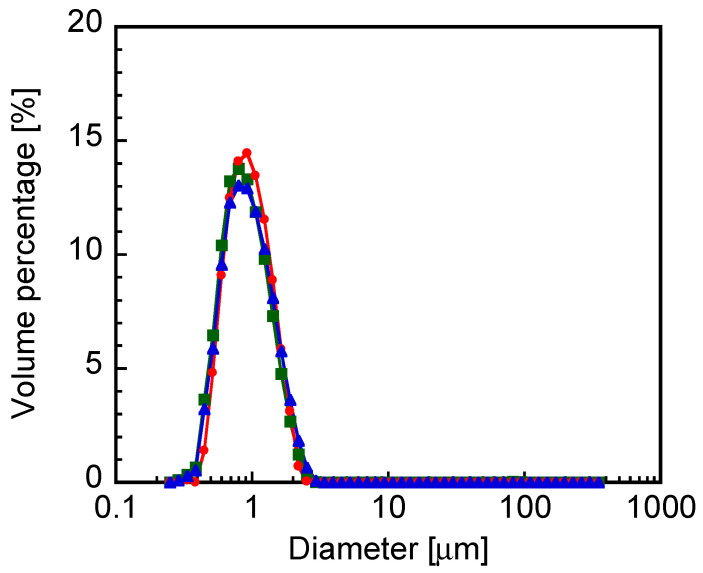
Particle diameter distributions of chitosan–oleic acid (CHI-OA) complex particles before (blue triangles) and after (red circles) mixing with 2 wt% agarose solution prior to gelation, and after gelation, followed by gel dissolution (green squares).

The CHI-OA complex particle dispersion was mixed with a 2 wt% AG aqueous solution in equal volumes to prepare a CHI-OA-AG dispersion containing 1 wt% AG. The resulting mixed dispersion was homogeneously opaque, with no visible aggregation or separation of the components. Figure 1 shows the particle diameter distribution of the CHI-OA complex particles in the CHI-OA and CHI-OA-AG dispersions. The particle diameter distributions were almost identical in the absence and presence of AG, with mean diameters of 0.86 and 0.87 μm, respectively. The diameter distribution of CHI-OA complex particles measured after dissolution of the CHI-OA-AG composite gel at 90 °C was also similar, with a mean diameter of 0.83 μm. These results indicate that the CHI-OA complex particles remained essentially unchanged during gel preparation and subsequent dissolution, suggesting that no significant aggregation or structural alteration occurred. In contrast, the ζ-potential of the CHI-OA particles decreased from +46 mV to +7.5 mV after melting the CHI-OA-AG gel and diluting the dispersion, even though the particle size remained almost unchanged. This result suggests that the electrokinetic properties of the CHI-OA complex particles were altered in the presence of agarose, which may indicate a weak physical association between the particles and agarose chains persisting after gel dissolution. Taken together, these results support that the CHI-OA complex particles were maintained as dispersed entities throughout gel formation and dissolution, while their interfacial electrokinetic properties may have been modified by the presence of agarose.

**Figure 2:**
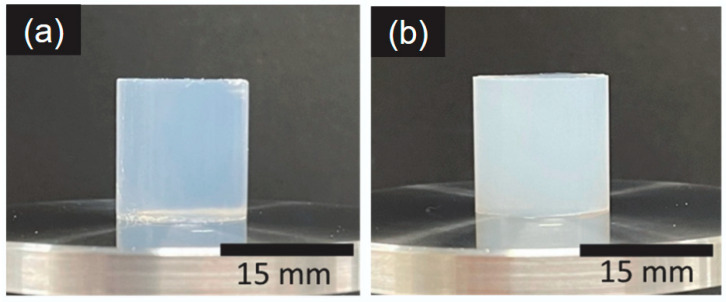
Appearance of 1 wt% AG gels in the (**a**) absence and (**b**) presence of CHI-OA complex particles. The gels were cut into cylindrical shapes; their diameter and height were both 15 mm.

**Figure 3:**
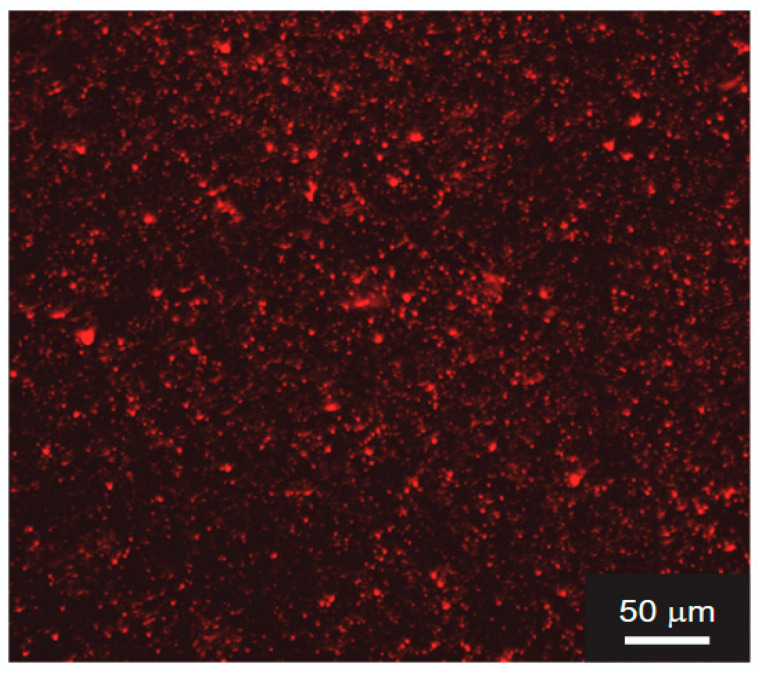
Confocal photomicrograph of chitosan-oleic acid-agarose (CHI-OA-AG) gel. CHI-OA complex particles were stained with Nile Red, a hydrophobic fluorescent dye.

Figure 2a,b show the appearance of the AG gel and CHI-OA-AG gel obtained by cooling a 1 wt% AG solution and a CHI-OA-AG dispersion containing 1 wt% AG at 4 °C. The AG gel was translucent, whereas the CHI-OA-AG gel was uniformly opaque, indicating reduced transparency due to the presence of CHI-OA complex particles. The CHI-OA complex particles were stained with Nile Red and observed using CLSM (Figure 3). Red fluorescent dots with submicron size were uniformly distributed throughout the gel, indicating homogeneous dispersion of the particles without significant aggregation. Furthermore, optical microscopy observations revealed that CHI-OA complex particles dispersed in the solution exhibited vibrational motion due to Brownian motion, whereas those incorporated within the gel remained stationary. These results suggest that CHI-OA complex particles are entrapped in the AG gel while maintaining their dispersed state.

To evaluate the storage stability of the CHI-OA-AG composite gel, samples prepared with 1 wt% AG were stored at 4 °C for 10 days. After storage, the gel largely retained its original morphology, with no observable particle sedimentation or localization within the gel matrix. The gel exhibited minimal syneresis, retaining 98% of its initial mass. In addition, the gel was melted in an acetate buffer at 90 °C, and the particle size distribution was measured by laser diffraction. A homogeneous dispersion with a mean particle diameter of 0.90 μm was obtained, indicating that the entrapped CHI-OA complex particles maintained their original size without aggregation. These results demonstrate that the CHI-OA-AG composite gel remains structurally stable during storage at 4 °C for at least 10 days.

### 2.2. Effect of the Addition of CHI-OA Complex Particles on the Physical Properties of AG Gel

**Figure 4:**
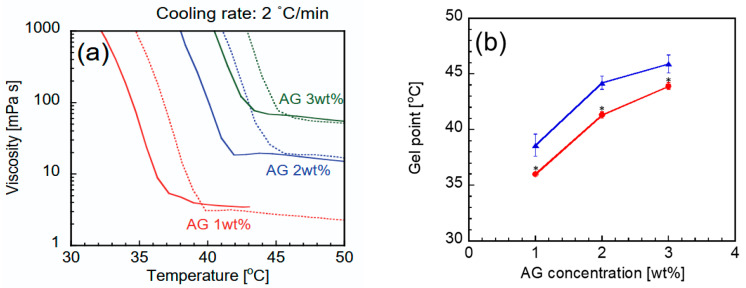
(**a**) Temperature dependency of the viscosities of AG solutions with different AG concentrations in the absence (dotted lines) and presence (solid lines) of CHI-OA complex particles. (**b**) The gel point of AG solutions with different AG concentrations in the absence (blue triangles) and presence (red circles) of CHI-OA complex particles. * indicates a statistically significant difference (*p* < 0.05) compared with AG gels without CHI-OA complex particles.

The viscosity changes in the CHI-OA-AG dispersion during the cooling process were measured to evaluate gel formation. Figure 4a shows the change in the apparent viscosity of CHI-OA-AG dispersion (AG concentration: 1, 2, and 3 wt%) during cooling from 50 °C at a rate of 2 °C/min. For all AG concentrations in the dispersion (sol) state, the viscosity-temperature curves were almost the same regardless of the presence or absence of CHI-OA complex particles, and no predominant change in AG sol viscosity was observed with the addition of CHI-OA complex particles. This suggests that there were no interactions affecting the viscosity of the CHI-OA complex particles or dissolved AG molecules in the sol state.

As shown in Figure 4a, the viscosity of all samples increased rapidly below a certain temperature during cooling. This rapid change in viscosity reflects the gelation of the AG sol. Figure 4b shows the gel point of each sample determined from the temperature-viscosity curve (Section 4.5 and Figure S1). The gel point was 2–3 °C lower in the presence of CHI-OA complex particles compared to that in their absence at the same AG concentration. Gelation of the AG solution during cooling is induced by the transition of coiled AG molecules in the sol to a helical form, followed by aggregation of the generated helix [29,31,32]. This coil–helix transition and helix aggregation are mainly caused by non-covalent interactions, including hydrogen bonds, which form cross-linking points in the AG gel [32,33,34]. The CHI-OA complex particles coexisting in the AG sol may physically inhibit helical aggregation, leading to a decrease in the gel point. Given that the size of the three-dimensional network structure of the AG gel is at the submicron level [35,36,37] and the diameters of the CHI-OA complex particles are of a similar or slightly larger order, physical or steric inhibition of AG gel formation by the CHI-OA complex particles is a plausible hypothesis. Although electrostatic and hydrophobic interactions can also influence gel formation, such interactions are likely limited in this system due to the neutral and hydrophilic nature of agarose, and the observed decrease in gel point suggests that attractive interactions between agarose and the particles are not dominant.

**Figure 5:**
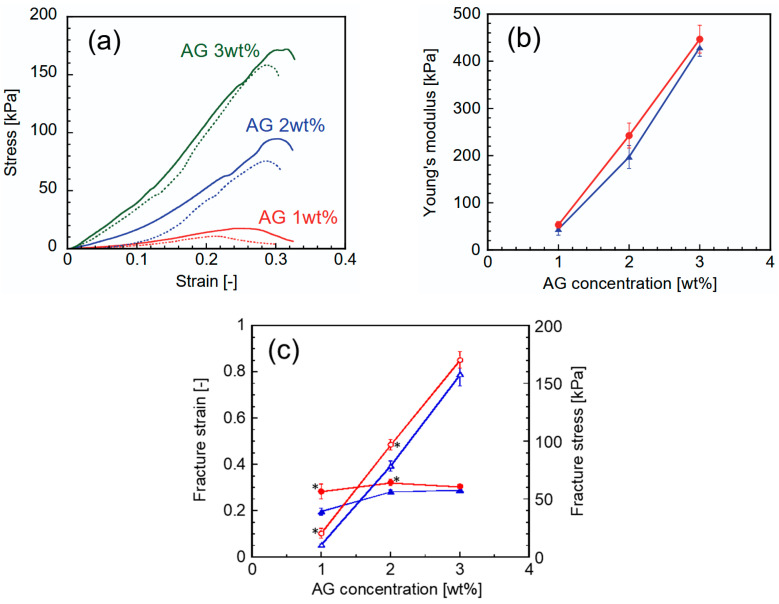
(**a**) Stress–strain curves of AG gels with different AG concentrations in the absence (dotted lines) and presence (solid lines) of CHI-OA complex particles. (**b**) Young’s modulus of AG gels in the absence (blue triangle) and presence (red circles) of CHI-OA complex particles. (**c**) The fracture strain (solid symbols) and fracture stress (open symbols) of AG gels as a function of AG concentration in the absence (blue triangles) and presence (red circles) of CHI-OA complex particles. The gel samples used for the compression test were cut into cylindrical shapes with the same diameter and height of 15 mm. * indicates a statistically significant difference (*p* < 0.05) compared with AG gels without CHI-OA complex particles.

Figure 5a shows the results of the compression tests. The tests were performed on 15 mm cylindrical gel samples. In all samples, the stress increased almost linearly with strain up to 0.1. Beyond this point, the stress showed a nonlinear increase with further strain. All samples exhibited fracture behavior, characterized by a sharp drop in stress within the strain range of 0.2–0.3, consistent with previous reports on agar gels [38,39]. Figure 5b shows the Young’s modulus *E* of each sample calculated from the data in the linear region of the stress–strain curves using Equation (3). The value of *E* increased slightly with increasing AG concentration. The values of the CHI-OA-AG gel were higher than those of the AG gel at the same AG concentration. The fracture strain and stress also significantly increased with the addition of CHI-OA complex particles, as shown in Figure 5c. Therefore, the addition of CHI-OA complex particles increases the strength of AG gels in both small and large deformation regions. The CHI-OA complex particles entrapped in the AG gel likely fill the voids in its three-dimensional network structure. The pore size of this network is on the order of several hundred nanometers [35,36,37]. A similar increase in rupture strength has been reported in emulsion gels, where small oil droplets are packed within the gel pores [40]. In addition, the physical properties of CHI-OA-AG gels showed a similar dependence on AG concentration as AG alone. This indicates that the gel strength can be controlled by adjusting the AG concentration.

### 2.3. In Vitro Digestive Properties of CHI-OA-AG Gels

Investigating the digestive properties of newly developed food materials provides important insights into the design of functional foods for individuals at various life stages, such as infants and the elderly. Therefore, we evaluated the gastric digestive properties of the CHI-OA-AG gels obtained in this study using in vitro digestion experiments with the FS method and the GDS [39,41]. The AG concentration of the gel samples used was 1 wt%, and gels molded into 5 mm squares were used for the experiments.

**Figure 6:**
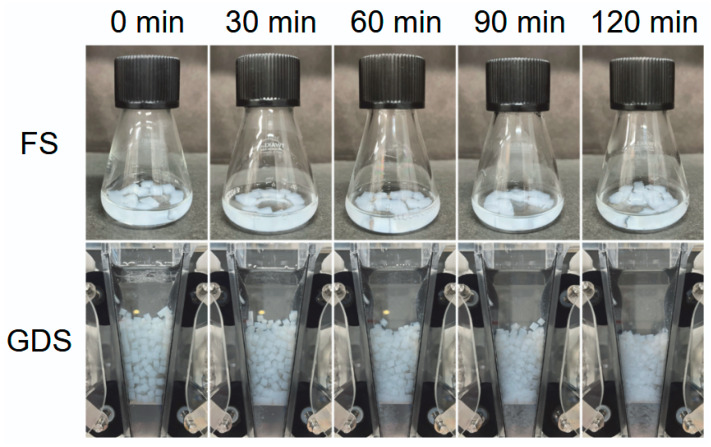
Snapshot photos of the digestion experiment of CHI-OA-AG gels using a flask-shaking equipment (FS) and a gastric digestion simulator (GDS). The gels (AG concentration: 1 wt%) were cut into 5 mm cubes and used for the experiments.

Figure 6 shows the appearance of the FS and GDS digestion test courses over time. After 120 min of FS, the sample retained its cubic gel shape as observed during the initial stage of digestion (top photographs in Figure 6). Compared to the gel immediately after preparation, the fracture strain and fracture stress decreased by 5.6% and 17.0%, respectively, indicating that the gel samples treated with the simulated digestive solution required less strain and stress to rupture. Although the gel samples did not undergo significant shape changes after FS, the CHI-OA-AG gels exhibited a slight tendency to collapse after treatment with the simulated digestive solution. This result suggests, consistent with previous studies [29,39,41], that FS does not produce experimental results that reflect the physical effects of gastric digestion.

**Figure 7:**
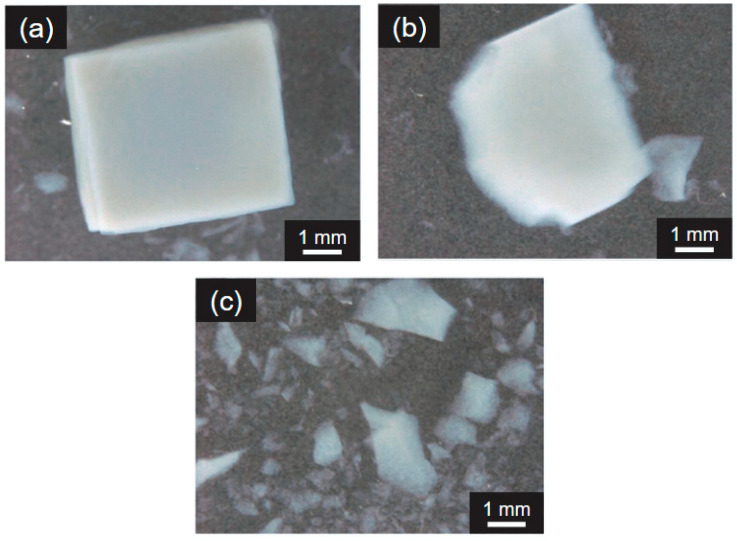
Typical shapes of the digested AG gels after 120 min in the GDS: (**a**) undamaged cubic gel; (**b**) partially damaged gels; (**c**) broken-down fragments with random shapes.

In contrast, during the GDS, it was observed that some of the gel was damaged. The packed volume of the gel in the GDS vessel decreased as the digestion time progressed (bottom photos in Figure 6). The typical appearance of the CHI-OA-AG gel recovered from the GDS vessel after 120 min of digestion is shown in Figure 7. In addition, some gels nearly retained their initial cubic shape (Figure 7a); others were damaged (Figure 7b); and small fragments (Figure 7c) were generated by gel disintegration, where parts of the gel were scraped off due to peristaltic mixing in the GDS. Given that the gel shape remained almost unchanged in the FS, it is possible that the GDS can better evaluate digestion behavior by accounting for physical changes, such as gel disintegration and fragmentation. Therefore, compared to FS, GDS is effective for assessing the digestion behavior, particularly concerning physical changes in the gel.

**Figure 8:**
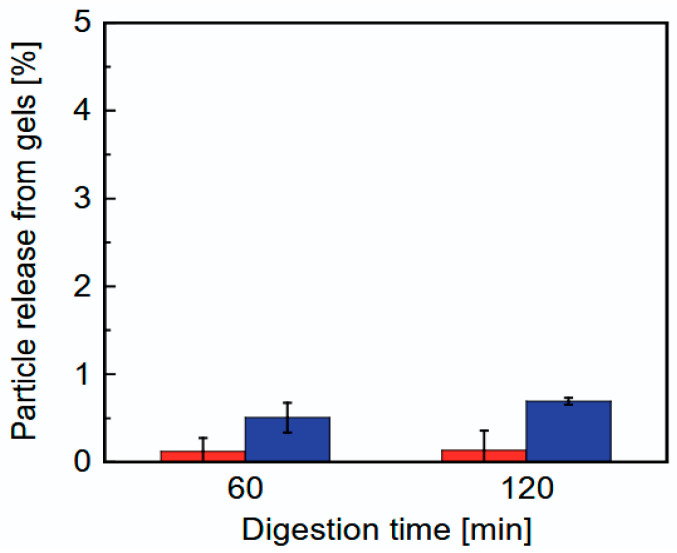
Particle release from gels into the digestion medium during gastric digestion experiments using a shaking flask (red) and a GDS (blue).

Furthermore, we investigated the release behavior of the CHI-OA complex particles from the CHI-OA-AG gel during an in vitro gastric digestion experiment. Figure 8 shows the percentages of CHI-OA complex particles released from the CHI-OA-AG gel into the simulated gastric fluid during the digestion experiments using FS and GDS. The release of complex particles from the gel was evaluated as a relative value based on changes in the turbidity of the simulated gastric fluid, with 100% representing the expected turbidity if all the CHI-OA complex particles were released into the simulated gastric fluid. In both the FS and GDS experiments, the percentage of CHI-OA complex particles released from the gel after 120 min of simulated gastric digestion was less than 1%, indicating that most of the CHI-OA complex particles were retained in the AG gel matrix.

When comparing the FS and GDS, no significant release of particles was observed in FS at 60 and 120 min. In contrast, particle release in GDS was slightly higher than in FS; however, the difference was not statistically significant (*p* = 0.06–0.08). This result suggests that the disintegration of the gel due to peristaltic motion in GDS may promote the release of complex particles, although the effect appears to be limited. In fact, in both digestion models, only a negligible amount of CHI-OA complex particles was released from the gels after 120 min. Compared to the release behavior of the oil phase from a 3 wt% agar gel encapsulating oil droplets (approximately 30% of the oil was released in 120 min) [41], the amount of CHI-OA complex particles released from the 1 wt% agar gel was very small.

In emulsion-containing agar gels, the oil droplets (20 μm) are much larger than the network structure of the agar gel matrix [41]. Therefore, it is believed that the gel matrix near the oil droplets is coarse, allowing the oil droplets to leak from the broken surface of the gel easily. In contrast, the CHI-OA complex particles in this study were comparable in size to the gel network structure and were encapsulated in AG gel matrices. Even when new cross-sections were created owing to gel disintegration by the peristaltic movement of the GDS, the particles remained trapped within the gel structure, making their release difficult.

This difference in the release behavior of the oil droplets and CHI-OA complex particles is notable when considering the digestion behavior of hydrophobic nutritional, functional ingredients encapsulated in agar- or AG-based gels. Hydrophobic nutrients added to the oil phase of an emulsion gel are released into the gastric juice along with the oil phase as the gel disintegrates during gastric digestion. In contrast, the hydrophobic nutrients encapsulated within the internal hydrophobic domains of the CHI-OA particles were scarcely released into the simulated gastric juice. They remained trapped in the gel fragments, undergoing downstream digestion in the small intestine. These distinct release modes suggest that such gels can be tailored to retain and deliver ingredients sensitive to the stomach’s low pH or designed to be released slowly in the small intestine. The low particle release during the gastric digestion phase supports this function by improving the retention of unstable bioactive compounds under gastric conditions. Such retention can be attributed to size exclusion effects, as the particle size is comparable to or larger than the pore size of the gel network, restricting particle mobility within the matrix. In addition, the retention of particles within the gel matrix may enable sustained release in the intestinal phase or potential delivery to the colon, although the detailed design of gel properties for these applications remains an important subject for future investigation.

### 2.4. Encapsulation of CUR into CHI-OA-AG Gels

Our previous studies [23,24,25,26] have demonstrated that CHI-OA complex particles possess a beneficial characteristic: the ability to encapsulate hydrophobic functional components. In this study, we encapsulated CUR, a hydrophobic nutritional ingredient, in the CHI-OA-AG gel described in the previous section. CUR was dissolved in ethanol with OA, which was then added dropwise to the CHI solution to form a CHI-OA complex particle dispersion encapsulating CUR. This dispersion was subsequently mixed with an aqueous AG solution, as described above, cooled, and gelatinized to produce a CHI-OA-AG gel containing CUR.

**Figure 9:**
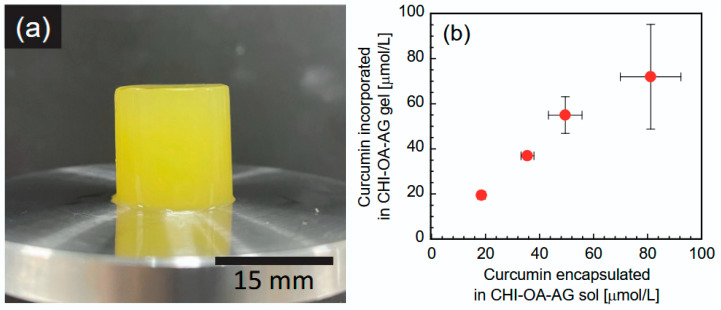
(**a**) Appearance of a CHI-OA-AG gel containing 72.0 M of curcumin (CUR) at an AG concentration of 1 wt%. (**b**) Effect of an added amount of CUR to the CHI-OA-AG dispersion (sol) on the incorporated amount of CUR in the CHI-OA-AG gel. AG concentration was 1 wt%.

Figure 9a shows the appearance of the CHI-OA-AG gel incorporating CUR (72.0 μmol/L CUR concentration in the gel). An intense yellow color derived from CUR was observed in the gel sample, indicating the absence of CUR aggregation, precipitation, or localization within the gel. Our previous report [23] suggested that CUR was incorporated into CHI-OA complex particles by partitioning them into aggregated OA domains within the particles, allowing them to maintain a stable dispersion. As the dispersion properties of the CHI-OA complex particles remained unchanged even after mixing with the AG solution (Figure 1), the CHI-OA composite particles were uniformly entrapped in the AG gel matrix, resulting in a highly homogeneous gel material. Furthermore, compression tests showed no significant differences in fracture strain or fracture stress between gels with and without CUR. These findings indicated that the addition of CUR did not affect the physical properties of the CHI-OA-AG gel under the conditions examined in this study.

Figure 9b shows the effect of the CUR content in the CHI-OA-AG dispersion on the incorporation of CUR into the CHI-OA-AG gel. The amount of CUR incorporated into the CHI-OA-AG gel increased proportionally with CUR content in the CHI-OA-AG dispersion before gelation. The incorporation efficiency was almost 100% within the range of CUR content examined. As reported in a previous study [23], the encapsulation efficiency of CUR into the CHI-OA complex particle dispersion was approximately 85% or higher, suggesting that 85% of the CUR used for the experiment was incorporated into the CHI-OA-CUR gel. This suggested that most of the CUR encapsulated in the CHI-OA complex particles remained effectively entrapped within the AG gel matrix. Although the loading capacity, i.e., the amount of CUR that can be loaded per unit carrier mass, is lower than that reported for some hydrogel-based delivery systems [42,43,44,45,46], previously reported encapsulation efficiencies for hydrophobic compounds typically range from approximately 50% to 95%, depending on the material and preparation conditions. In the present system, the overall encapsulation efficiency remained at a similarly high level, as most of the CUR encapsulated in the CHI-OA particles was retained during gel formation. These results suggest that the proposed system enables efficient retention of hydrophobic compounds during the transition from a particle dispersion to a gel structure, with minimal loss during processing. Since the CHI-OA complex particle dispersion can be concentrated while retaining CUR via evaporation of the dispersing medium [23], further improvement in loading capacity may be expected by using concentrated CHI-OA complex particles for gel preparation. Improving the loading ability of the hydrophobic components is an important challenge that we should investigate in the future.

Overall, these results demonstrated that the CHI-OA-AG gel can successfully encapsulate CUR. Further research, including the evaluation of the bioaccessibility and bioavailability [3,10] of encapsulated hydrophobic bioactives, may enhance the development of new food materials aimed at achieving efficient delivery of various nutritional components to the human body.

## 3. Conclusions

The CHI-OA particles were stably incorporated in the AG gel matrix while maintaining their original features. The obtained CHI-OA-AG composite gels maintained their macroscopic appearance and structural integrity at 4 °C for at least 10 days, suggesting their potential stability during storage. The incorporation of the CHI-OA particles lowered the gelation point and modified the fracture strain and stress of the gel. In vitro gastric digestion tests using GDS with simulated peristaltic motion revealed that the CHI-OA-AG gels experienced disintegration during the gastric digestion phase, although the release of particles from the fractured gel surface was minimal. This behavior, distinct from conventional emulsion gels that exhibit pronounced oil-phase release under gastric conditions, suggests that the structural confinement of particles within the gel matrix contributes to their retention and highlights its potential as a novel nutrition carrier enabling sustained release of hydrophobic nutrients in the small intestine and colon.

In addition, CHI-OA-AG gels containing CUR were successfully prepared using CUR encapsulated in CHI-OA complex particles, with CUR uniformly dispersed and retained within the AG gel without visible phase separation. These findings suggest that CHI-OA-AG gels show potential as delivery systems for water-insoluble hydrophobic nutrients. Further investigation of their in vitro digestion behaviors, including bioaccessibility and bioavailability, along with optimization of the gel preparation process, could broaden their applications as innovative food materials. Such characteristics, combining effective carrier functionality with compatibility for incorporation into structured gel matrices, may be particularly advantageous for emerging applications such as personalized nutrition and 3D printed food systems.

## 4. Materials and Methods

### 4.1. Chemicals

CHI (Chitosan 10^®^, degree of deacetylation = 85%, viscometric average molecular weight = 150,000) [47], OA, AG (Agarose I), CUR, ethanol, methanol, acetic acid, Nile Red, sodium hydroxide, potassium chloride (KCl), sodium chloride (NaCl), hydrochloric acid (HCl), potassium dihydrogen phosphate (KH_2_PO_4_), and calcium chloride dihydrate (CaCl_2_(H_2_O)_2_) were purchased from FUJIFILM Wako Pure Chemical Corporation, Osaka, Japan. Magnesium chloride hexahydrate (MgCl_2_(H_2_O)_6_) was purchased from Kato Chemical, Co., Inc., Tokyo, Japan. Porcine pepsin, ammonium carbonate ((NH_4_)_2_CO_3_), and sodium bicarbonate (NaHCO_3_) were purchased from Sigma-Aldrich Co., St. Louis, MO, USA. All chemicals were used as purchased without further purification. Ultrapure water used throughout the experiments was generated using a Direct-Q water purification system (Merck Millipore Corporation, Billerica, MA, USA), with a resistivity of 18.2 MΩ∙cm.

### 4.2. Preparation of CHI-OA-AG Gels

CHI-OA complex particles were prepared according to our previous report [23]. Briefly, 2 mL of ethanol containing 0.0525 M OA was added dropwise to 20 mL of the CHI solution (5 g/L, pH 5.0) under magnetic stirring at 400 rpm and room temperature (22–26 °C). The molar mixing ratio of OA and CHI was 0.2 [23]. The mixture was further stirred for 30 min under the same conditions and then centrifuged at 2000 rpm and 25 °C for 10 min to remove coarse aggregates in the sample.

AG was dissolved in water at 90 °C to obtain 2, 4, and 6 wt% AG solutions. The AG solution was mixed with an equal volume of CHI-OA complex particle dispersion at 90 °C. The resulting CHI-OA-AG dispersion was placed in a 15 mm diameter syringe and cooled at 4 °C for 18 h to formulate CHI-OA-AG gels.

To encapsulate CUR in the CHI-OA-AG gel, CUR was added to ethanol with OA to prepare the CHI-OA complex particle dispersion. The resultant dispersion was mixed with AG solution and cooled at 4 °C for 18 h to obtain CHI-OA-AG gels encapsulating CUR.

### 4.3. Measurement of Particle Diameter Distributions

Particle size distributions of the CHI-OA complex particles were determined using a laser diffraction analyzer (SALD-200V ER, Shimadzu Corporation, Kyoto, Japan). Prior to measurement, each sample was appropriately diluted with water to achieve suitable turbidity; dilution was carried out at 22–26 °C for CHI-OA dispersions and at 50 °C for CHI-OA-AG mixtures.

### 4.4. Measurement of ζ-Potential

The ζ-potential of CHI-OA complex particles was measured using a laser Doppler electrophoretic light scattering analyzer (Zetasizer Nano Z, Malvern Instruments, Worcestershire, UK). Samples were diluted 100-fold to achieve an appropriate scattering intensity. Dilution was performed at 22–26 °C for CHI-OA dispersions and at 50 °C for CHI-OA-AG mixtures.

### 4.5. Confocal Laser Scanning Microscopy (CLSM)

The dispersion state of CHI-OA complex particles in CHI-OA-AG gels was observed using an upright microscope (ECLIPSE Ni-E, Nikon Corporation, Tokyo, Japan) equipped with a confocal unit (AX R, Nikon Corporation, Tokyo, Japan). The CHI-OA complex particles were stained with Nile Red, which was added to the ethanol solution containing OA during the preparation described in Section 4.2. Observations were performed at an excitation wavelength of 561 nm.

### 4.6. Evaluation of Gel Stability

AG gels and CHI-OA-AG gels were prepared in cylindrical shapes with diameters and heights of 15 mm. Each gel sample was placed in a glass weighing bottle (inner diameter: 30 mm) and incubated at 4 °C for 10 days using a cooling incubator (i-Cube FCS280, As One Corporation, Tokyo, Japan). Gel weights were measured before and after incubation to evaluate syneresis. The particle size distribution of CHI-OA complex particles was determined as described in Section 4.3 after dissolving the gel samples in acetate buffer (pH 5.0) at 90 °C for 15 min.

### 4.7. Viscosity Measurement

The viscosities of the AG solutions and CHI-OA-AG mixtures were measured using a vibrational viscometer (SV-10, A&D, Tokyo, Japan) equipped with a sample chamber and water circulator to control the sample temperature. The cooling rate of the samples from 50 to 30 °C was 2 °C/min. The gel point of each sample was determined from the temperature-viscosity profiles, as shown in Figure S1 (Supplementary Material).

### 4.8. Compression Test of Gel Samples

The gel samples were formulated into cylindrical shapes with diameters and heights of 15 mm, respectively. The compression tests of the gel samples were performed using a tabletop universal testing machine (MCT-1150; A&D Co., Ltd., Tokyo, Japan). The cylindrical gel sample was axially compressed between two plungers with a diameter of 60 mm at a compression rate of 0.5 mm/s until the gel was fractured. The load (*F*) and displacement (Δ*L*) were measured, and the stress (*σ*) and strain (*ε*) were calculated as follows:

*σ* = *F*/*A*_i_ (1)

*ε* = Δ*L*/*L*_i_ (2)

where *A*_i_ and *L*_i_ are the initial cross-sectional area and initial height of the cylindrical gel sample, respectively. The Young’s modulus (*E*) was determined from the initial linear region of the stress–strain curves using the following equation:

*E* = *σ*/*ε* (3)

### 4.9. In Vitro Digestion Experiments

#### 4.9.1. Preparation of Simulated Digestive Fluids

An in vitro gastrointestinal digestion system including oral and gastric phases was constructed in accordance with the standardized INFOGEST protocol [30], with minor modifications. The simulated salivary fluid was prepared as an electrolyte solution with a defined composition, comprising KCl (15.1 mmol/L), KH_2_PO_4_ (3.7 mmol/L), NaHCO_3_ (13.6 mmol/L), MgCl_2_·6H_2_O (0.15 mmol/L), (NH_4_)_2_CO_3_ (0.06 mmol/L), HCl (1.1 mmol/L), and CaCl_2_·2H_2_O (1.5 mmol/L), with a total volume of 1.61 mL. Salivary amylase was not included, as the samples did not contain starch.

For the gastric phase, the simulated gastric fluid was prepared as an electrolyte mixture (total volume: 3.202 mL) containing KCl (6.9 mmol/L), KH_2_PO_4_ (0.9 mmol/L), NaHCO_3_ (25 mmol/L), MgCl_2_·6H_2_O (0.12 mmol/L), (NH_4_)_2_CO_3_ (0.5 mmol/L), HCl (15.6 mmol/L), CaCl_2_·2H_2_O (0.15 mmol/L), and NaCl (47.2 mmol/L). Pepsin was subsequently introduced into the digestion system to achieve a final activity of 2000 U/mL.

#### 4.9.2. Preparation of CHI-OA-AG Gel for In Vitro Digestion Experiments

A CHI-OA-AG dispersion containing 1 wt% AG was prepared as described in Section 4.2. The dispersion was poured into a polystyrene tray to obtain a liquid depth of 5 mm and then cooled at 4 °C for 18 h to form a CHI-OA-AG gel. The resulting gel plate was cut into 5 mm cubes using a multi-blade cutter [48]. These cubic agar gels were used for the in vitro digestion experiments.

#### 4.9.3. In Vitro Digestion Using the Flask-Shaking (FS) Method

Before conducting each FS experiment, a simplified in vitro oral digestion step was carried out. In this procedure, 2 g of CHI-OA-AG gel cubes were mixed with 2 mL of simulated salivary fluid in a 30 mL Erlenmeyer flask, followed by incubation at 37 °C under shaking conditions (120 strokes/min) for 2 min. Subsequently, the FS-based gastric digestion was initiated by adding 4 mL of simulated gastric fluid (pH 3.0) to the oral digestion mixture. The resulting system was further incubated at 37 °C with shaking at 120 strokes/min for 120 min.

#### 4.9.4. In Vitro Digestion Using Gastric Digestion Simulator (GDS)

**Figure 10:**
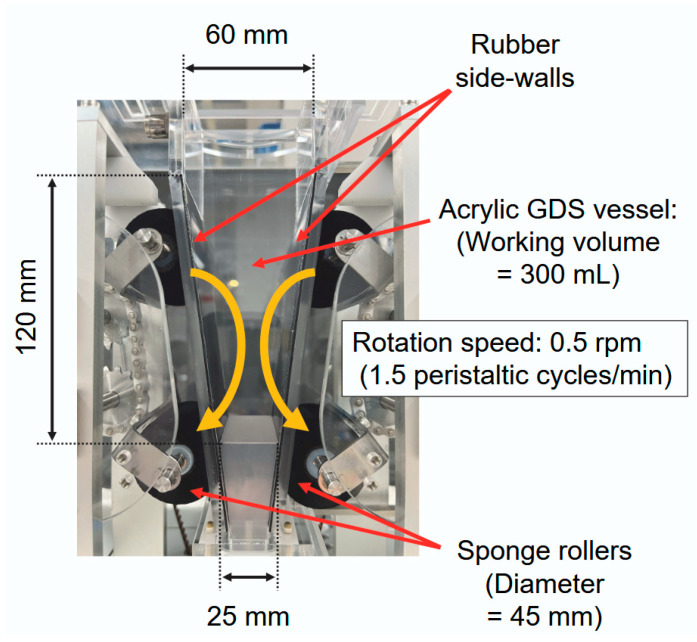
Gastric digestion simulator (GDS) used in this study. Yellow arrows indicate the direction of roller rotation.

Figure 10 shows a schematic of the GDS (EP-Tech, Hitachi, Japan) used in this study. An acrylic GDS vessel with a working volume of 300 mL was used. Simulated gastric peristalsis was generated by the rotational motion of three sponge rollers positioned along the rubber sidewalls of the GDS vessel. These rollers moved downward toward the bottom of the GDS vessel, thereby simulating the function of the pylorus. According to a previous report on gastric peristalsis in human adults [49], the frequency of peristaltic motion was adjusted to 1.5 cycles/min of antrum contraction waves. Throughout the experiments, the internal temperature of the GDS vessel was maintained at 37 °C using a heating unit equipped with the GDS instrument.

In addition, before in vitro gastric digestion, 75 g of 5 mm CHI-OA-AG gel cubes were mixed with 75 mL of simulated saliva (pH 7.0) and shaken at 120 strokes/min and 37 °C for 2 min to simulate oral digestion. The mixture containing the CHI-OA-AG gels was immediately transferred into the GDS vessel filled with simulated gastric fluid (150 mL, pH 3.0). Notably, each in vitro gastric digestion experiment was conducted for 120 min. The transparent acrylic windows of the GDS vessel enabled real-time monitoring of gel sample disintegration during digestion. Aliquots of digested samples were periodically obtained from the GDS vessel. After digestion, the gel shape was observed using a digital microscope consisting of a zoom lens (TS-93005, Sugitoh Co., Ltd., Tokyo, Japan) and a CCD camera (STC-TC-202USB-AS, Omron Sentech Co., Ltd., Ebina, Japan). The optical density of the liquid phase in the digest was measured at 660 nm using an ultraviolet (UV)–visible spectrophotometer (UV-1800, Shimadzu Corporation, Kyoto, Japan) after separating the gels by centrifugation.

### 4.10. Determination of CUR Content

An aliquot (0.15 mL) of the CHI-OA particle suspension containing CUR was withdrawn and mixed with 1.35 mL of methanol. The resulting mixture was left at room temperature (22–26 °C) for 5 min to ensure complete extraction of CUR and separation of the CHI precipitate. The sample was then centrifuged at 2500 rpm for 15 min using a centrifuge (Micro Six MS-1, As One Corporation, Tokyo, Japan). Following centrifugation, the supernatant was filtered through a syringe-connected membrane filter (0.45 μm pore size; Dismic 13HP045AN, Advantec Toyo Kaisha, Ltd., Tokyo, Japan) to remove any precipitate. The absorbance of the filtrate was measured at 420 nm using a UV–1800 spectrophotometer. The CUR concentration was quantified based on a calibration curve prepared preliminarily using methanol-dissolved CUR.

For the measurement of CUR content in the CHI-OA-AG gel, 0.15 g of the gel was crushed into small fragments (<1 mm), weighed, and mixed with 1.35 mL of methanol. CUR incorporated in the gel was extracted into the liquid phase of the sample by sonication at room temperature (22–26 °C) for 10 min in a sonic bath (D-SONiC US-350S, Sansyo Co., Ltd., Tokyo, Japan). Subsequent centrifugation, filtration, and absorbance measurements were performed as described in this section.

### 4.11. Statistical Analysis

All experiments were performed with at least three independent samples (*n* ≥ 3). Data are presented as mean ± standard deviation (SD), and error bars in figures represent SD. Statistical differences between two datasets were evaluated using Welch’s *t*-test (i.e., without assuming equal variances) in KaleidaGraph software (Version 4.01, Synergy Software, Reading, PA, USA). Differences were considered statistically significant at *p* < 0.05.

## Data Availability

Data will be made available on request.

## Supplementary Materials

The following supporting information can be downloaded at: https://www.mdpi.com/article/10.3390/gels12050374/s1, Figure S1: Determination of gel point by viscosity measurement. The gel point is the characteristic temperature at which the viscosity rapidly increases during cooling. In this study, the temperature at the intersection of straight lines A and B in the figure was defined as the gel point.
