# Supplementary material for: Formulation of Agarose Gels Containing Chitosan-Oleic Acid Complex Particles and Their Physical and In Vitro Digestion Properties
Source: Gels. 2026 Apr 29;12(5):374. doi: 10.3390/gels12050374 (PMC13205382; doi:10.3390/gels12050374)
Supplement: Supplementary file 1 [file gels-12-00374-s001.zip › gels-4216080-supplementary.pdf]

Supplementary material

Formulation of Agarose Gels Containing Chitosan-Oleic Acid Complex Particles and Their Physical and *In Vitro* Digestion Properties

Takashi Kuroiwa, Tsukasa Kikuchihara, Kana Kanemitsu, Airi Kato

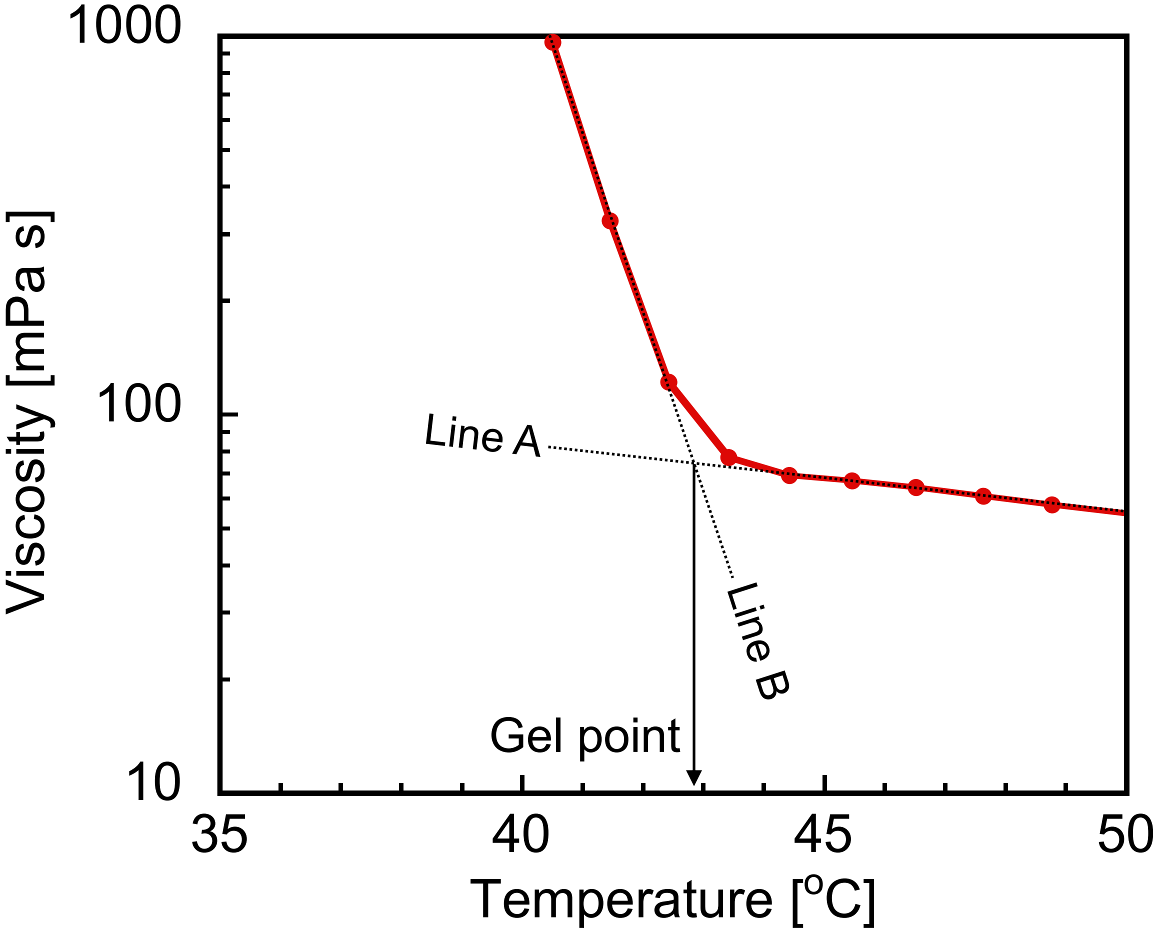

Figure S1. Determination of gel point by viscosity measurement. The gel point is the characteristic temperature at which the viscosity rapidly increases during cooling. In this study, the temperature at the intersection of straight lines A and B in the figure was defined as the gel point.
